# Effects of Species Invasion and Inundation on the Collembola Community in Coastal Mudflat Wetland from the Perspective of Functional Traits

**DOI:** 10.3390/insects14020210

**Published:** 2023-02-19

**Authors:** Jing-Yang Li, Yun-Xia Gao, Chun-Yang Li, Ya-Li Jin, Si-Qi Yang, Jian-Hong Xia, Yun-Fei Zhang, Yun Bu, Kai Li

**Affiliations:** 1 School of Life Sciences, East China Normal University, Shanghai 200241, China; 2 Shanghai Xi Jiao School, Shanghai 200335, China; 3 Shanghai Natural History Museum, Shanghai Science & Technology Museum, Shanghai 200041, China

**Keywords:** coastal mudflat wetland, springtail, functional trait, vegetation, tidal flat

## Abstract

**Simple Summary:**

The habitat of wetlands, especially coastal mudflat wetlands, has been seriously threatened by species invasion in recent years, as globalization has progressed worldwide. In this study, we compared the invasive species *Spartina alterniflora* with the native species *Phragmites australis* to reveal the effects of invasive plants on species diversity and functional traits of Collembola and explore the adaptation patterns of wetland Collembola in various vegetation and soil depths. We reveal how invasive plants affect coastal mudflat wetlands and offer a reference for conserving wetlands from the perspective of soil fauna.

**Abstract:**

The group of soil arthropods known as Collembola is characterized by its abundance and sensitivity to environmental changes. They are ideal an species for soil indicators. In order to clarify the effects of species invasion and inundation on the Collembola community in coastal mudflat wetlands, the correlation between the collembolan functional traits and environmental factors was studied in Shanghai Jiuduansha Wetland National Nature Reserve for the first time. Five sample plots, including three vegetations—*Spartina alterniflora* (an invasive species), *Phragmites australis*, and *Zizania latifolia*—were set up following the differences in vegetation types and between high and low tidal flats. Data on the diversity of the Collembolan species and their functional traits were collected and combined with the soil physicochemical properties and vegetation environment factors in different tidal flats. The key findings and conclusions of the study are as follows: a total of 18 species, four families, and three orders make up the obtained Collembola, two species of *Proisotoma* are dominant species that account for 49.59% and 24.91% of the total, respectively. The maintenance of the species diversity of Collembola is disturbed by the higher conversion efficiency of *Spartina alterniflora* rather than *Phragmites australis* with lower organic carbon (C) content and higher total nitrogen (N) content. The primary environmental variables influencing species distribution were the C/N ratio, total N, and bulk soil density. The bulk density of the soil impacts the movement and dispersal of the functional traits. The depth of the soil layer is related to the functional traits of the sensory ability. The analysis of the functional traits and environment is fairly helpful in exploring how species respond to their environment and offers a better explanation for the habitat selection of Collembola.

## 1. Introduction

Wetlands are one of the most significant and endangered ecosystems in the world. They are essential for biodiversity conservation, flood management, shoreline stabilization, and climate change mitigation and adaptation [1]. The influence of human activity on wetlands is gradually increasing due to the rapid economic development in the eastern coastal areas of China. The extent of coastal wetlands has significantly decreased, and the diversity of wetland species has rapidly degraded, especially in recent years due to species invasion and coastal breakwater construction [2,3,4]. Artificial reclamation and biological invasion by *Spartina alterniflora* are severe issues that need to be addressed immediately. Biodiversity loss, dwindling biological resources, and reduced areas are issues the eastern coastal wetlands must tackle [5]. Investigating how changing vegetation affects biomes is urgently needed.

Collembola is one of the most abundant and diverse soil microarthropods [6] and is sensitive to modifications in the soil environment [7]. Therefore, they are extremely vulnerable to environmental issues, such as habitat fragmentation or air pollution [8,9,10,11,12]. Collembola communities may be impacted, for example, by unusual wind damage and modifications to leaf litter areas in the forest [13,14]. Meanwhile, alterations in soil conductivity, organic matter, and soil pH caused by human activities can impact the spread of Collembola communities [15]. The findings of a study on the effects of seawater on the abundance and diversity of the grassland Collembola community in a coastal area indicate that the long-term effects of seawater had a severe negative impact on the abundance of and diversity of the springtail community, but the springtail community in the grassland immediately affected by seawater was hardly affected by such a negative impact. Even for some species, the short-term impact of seawater has a positive effect on their ability to proliferate [16]. The Collembola community can also prove the negative influence of the degradation of wetlands [17]. Furthermore, invasive plants, such as *Solidago canadensis* L., can alter the physicochemical properties of the soil, particularly the NH_4_-N, which in turn alters the Collembola community structure [18]. Nitrogen deposition can also affect the vertical distribution of the different habits of Collembola [19]. In general, springtails are an ideal group of soil animals to analyze the environment.

The research on functional traits has attracted extensive attention in the research of soil animal ecology because it can intuitively reflect the adaptation mechanism of organisms to the environment [20]. It was found that a large body size is more adaptable to severe climate change in high-latitude areas, but there is no significant impact of climate change on the Collembola community based on species classification [21]. Differentiating between high and low tidal flats and examining functional trait-environment interactions rather than simple species-environment relationships will uncover a specific adaptive mechanism for the variation of the tidal zone in environmentally unfavorable salt marsh wetlands [22]. Similarly, a functional traits analysis helps identify the influence of abiotic parameters [23] and reveals deeper patterns when examining issues, such as the impact of various land use types and environmental disturbance [24]. The diversity analysis of the springtail functional traits is an ideal technique for exploring ecological issues and predicting species communities in a specific environment. However, research on the functional traits of Collembola associated with the invasion of exotic plants still need to be studied.

In order to clarify the effects of species invasion and inundation on the Collembola community in coastal mudflat wetlands, this research was conducted on an offshore tidal flat wetland in the Yangtze River Delta of China called the Jiuduansha wetland. We explored the distribution and functional traits of the springtail community and its environmental responsibility model using the Collembola functional trait diversity research method. As the main environmental measurement factors, we selected crucial wetland environmental factors, such as the vegetation type, the difference between high and low tidal flats, soil pH, and soil C/N ratio. We speculate that *Spartina alterniflora*, an invasive plant, and seawater flooding will have a significant impact on the springtail community.

## 2. Materials and Methods

### 2.1. Study Sites and Experimental Design

**Figure 1:**
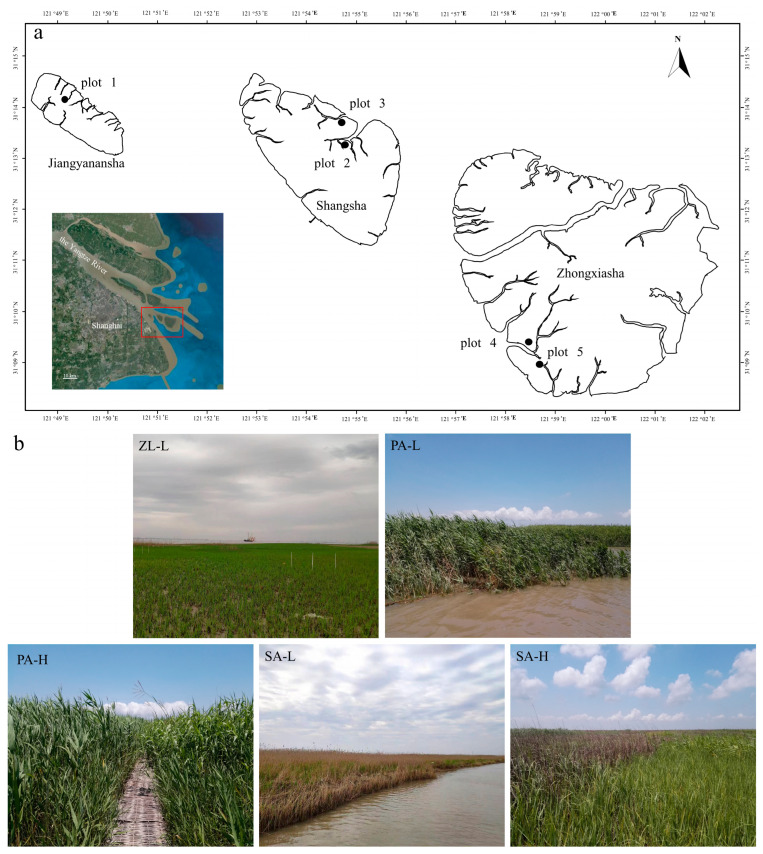
Schematic map of the studied sites in Jiuduansha wetland (**a**) and sample plots (**b**). See Table 1 for codes of the plots. The red frame shows the location of Jiuduansha wetland at the intersection of the Yangtze River Estuary.

The study was carried out at the Jiuduansha Wetland National Nature Reserve in Shanghai, China (122°46′~122°15′ E, 31°03′~31°17′ N) (Figure 1a). It is located at the intersection of the Yangtze River Estuary in East China. It has an average annual temperature of 15.7 °C and an average annual precipitation of 1143 mm [25]. The Jiangyanansha, Shangsha, and Zhongxiasha shoals make up the study site. When the tide rises in Jiangyanansha, the area with the lowest topography, the entire shoal will be submerged by seawater. Shangsha and Zhongxiasha contain several high tidal flats not permanently submerged by seawater due to their high topography [26,27]. *Phragmites australis*, *Scirpus Mariqueter*, and *Zizania latifolia* are the dominant indigenous plants. During these years, *Spartina alterniflora*, an invasive species, has spread over Zhongxiasha [28,29,30].

We designed five plots based on the differences in vegetation and the height of the tidal flats (Table 1, Figure 1b). Each plot contains three randomly selected sample sites with an area of 10 × 10 m, and each sample had three groups of repeats. Sample sites must be spaced apart by at least 30 m to prevent interference.

### 2.2. Collection and Treatment of the Samples

Soil samples were collected in the spring, summer, autumn, and winter between June 2018 and April 2019 using a sample corer with a 5 cm diameter and a 15 cm length. Each sample was divided into three layers (upper: 0–5 cm, middle: 5–10 cm, and lower: 10–15 cm). The samples were separately packaged and carried to the laboratory for treatment. The soil samples were treated using Tullgren’s funnel method [31] and the soil animals were fixed at 75% alcohol. All Collembola specimens were sorted out, mounted on slides, and identified at the species and genus levels by keys in monographs or articles [32,33,34,35,36]. Some colleagues working on the taxonomy of collembolan also gave their help. The Collembola from winter were less than 10 individuals, so they were not taken into account in the statistical analysis. A total of 135 soil samples were taken for faunal extraction (Table S1).

Seven response traits were chosen to identify the most likely factor affecting the distribution of Collembola in the environment, taking into account other studies related to the functional traits of Collembola [37,38,39,40,41,42]. The body size, body length, and furcula length influence the mobility and distribution capacity of the Collembola. The number of eyes, PAO, and length of the antennae, which are all related to sensory ability, directly impact how sensitive Collembola are to changes in their environment. Except for the dispersal ability of the collembola, the sensory ability of the Collembola is also associated with the distribution of the Collembola [24]. Body length and antenna length were measured under a microscope, and measurements of the specimen’s body size, furcula, eyes, and PAO were obtained (Table 2).

Each plot with 3 replicates soil samples were collected for soil analysis, following the soil environmental monitoring technical specification HJ/T 166 in China for the indoor assessment of soil physicochemical characteristics. Soil bulk density was collected by 100 cm^3^ cutting rings. Parts of the samples were air-dried and passed through a 2 mm sieve after removing the surface impurities and then used to determine the pH, total nitrogen, total phosphorus, and total organic carbon. The water content in the soil was determined by oven drying the soil at 105 °C for 12 h and measuring the mass of the soil before and after. The volume of cutting rings and the water content of the soil were used to calculate the bulk density. Potentiometry was used to measure the pH of the soil (Lei-ci PHS-3C, Shanghai REX Instrument Factory, Shanghai, China). Automatic discontinuous analyzers were used to test the total N and total P (SmartChem 200, AMS Alliance, Roma, Italy). We calculated the amount of organic C content in a total organic C analyzer (Model: Vario TOC, Elementar Analysensysteme GmbH, Langenselbold, Germany).

### 2.3. Statistical Analyses

The diversity of Collembola communities was calculated using the Shannon–Wiener diversity index (H), Margalef abundance index (D), Simpson dominance index (C), and Pielou evenness index (J) [43] as follows:

Shannon–Wiener diversity index:

H = −∑ni/Nln(ni/N)

where ni is the number of individuals in the ith group; N is the total number of individuals in all groups.

Pielou evenness index:

J = H/lnS

where S is the number of groups.

Simpson dominance index:

C = ∑Pi^2^

where Pi = ni/N.

Margalef abundance index:

D = (S − 1)/lnN

One-way analysis of variance was used to compare the characteristics of the species diversity of the springtail community and the variations in vegetation types and sites. The least significant difference (LSD) procedure and contrasts with a probability level of 0.05 were then used to identify the significant differences between treatment effects. The relationship between the springtail community and environmental conditions was examined using a Pearson correlation analysis (two-tailed test). The impact of various environments on the key groups in the springtail community was examined using a principal component analysis (PCA). IBM Statistical Package for Social Sciences 23.0 was used to conduct the statistical analysis. Figures were made using GraphPad Prism 7.

The environmental factors and springtail communities were analyzed using a de-trended correspondence analysis (DCA) and canonical correlation analysis (CCA) using CANOCO for Windows 4.5 ecological data multivariate statistical analysis software, and the corresponding Monte Carlo test was performed (499 times, *p* < 0.05). We used CanoDraw for Windows to draw the CCA ordering diagram. Environmental factors and springtail communities were subjected to DCA and linear model-redundancy analysis (RDA), and the Monte Carlo test was conducted as a result [44]. CanoDraw for Windows was used to create the RDA sequencing.

## 3. Results

### 3.1. Physicochemical Properties of the Soil in the Different Plots

According to the analysis, some of the physicochemical properties of soil in some of the five plots differed significantly from the others (Table 3). The *Phragmites australis* high-tide flat plot (PA-H), with a water content of 26.44%, was the highest. *Phragmites australis* high-tide flat plot (PA-H) and *Spartina alterniflora* low-tide flat plot (SA-L) had significantly higher bulk densities than *Phragmites australis* low-tide flat plot (PA-L) (*p* < 0.05). The soils of the five sites were all alkaline, and the pH values of the *Phragmites australis* high-tide flat plot (PA-H) and *Spartina alterniflora* high-tide flat plot (SA-H) were significantly higher than those of the *Zizania latifolia* plots (ZL-L) (*p* < 0.05). *Zizania latifolia* plots (ZL-L) had the highest total P content (0.56 g/kg), which was significantly greater than *Spartina alterniflora* high-tide flat plot (SA-H) (*p* < 0.05). Although there was no significant difference between the plots regarding organic C, the PA-L and PA-H values were higher in the plots where *Phragmites australis* was the dominant vegetation. The highest C/N ratio was observed in the *Phragmites australis* high-tide flat plot (PA-H), while the lowest C/N ratio was found in the *Spartina alterniflora* high-tide flat plot (SA-H). However, there was no significant difference in the C/N ratio among the sample plots (SA-H). The results of the two-factor analysis indicated pH and TOC are significantly affected by tidal flat, BD are significantly affected by the interaction of the tidal flat and plant (Table S2).

### 3.2. Community Composition of Collembola

Totally, 851 springtails from five plots were collected for this study. Following the mounting and identifying of all specimens, 18 species belonging to three orders and four families were determined. The two major species, accounting for 49.59% and 24.91% of the total, respectively, are *Proisotoma minuta* (Tullberg, 1871) and *Proisotoma* sp. (individuals in the total number exceed 10%). The following common taxa make up 1–10% of the total: *Subisotoma* sp., *Folsomides* sp. 1, *Coecobrya pani* Xu, Yu & Zhang, 2012, and *Sphaeridia* sp., accounting for 7.64%, 5.88%, 4.35%, and 5.17% of the total, respectively. Together they account for 20.33% of the total. The remaining rare species (<1% of the entire population) account for only 5.17% of the total (Table 4).

### 3.3. Response of Collembola Species Diversity to the Environment

**Figure 2:**
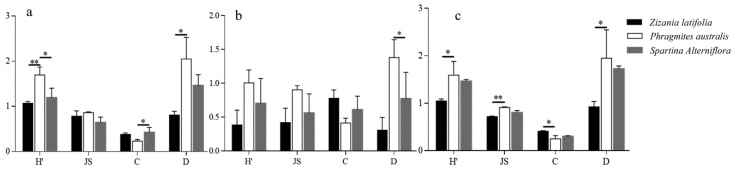
Comparison of the diversity indices of the different vegetation in (**a**) spring, (**b**) summer, and (**c**) autumn. H’ = Shannon–Wiener index, JS = Pielou’s evenness index, C = Simpson dominance index, and D = Margalef abundance index. The values are the means ± SE. * indicates the level of significance. * = significant (*p* < 0.05), ** = extremely significant (*p* < 0.01).

Evaluating the species diversity of the community of springtails in various vegetation types and seasons, the findings revealed that SA-L (*p* < 0.05) and SA-H (*p* < 0.05) had a significantly lower Shannon–Wiener diversity index than PA-L and PA-H. The findings revealed that ZL-L (*p* < 0.01) had an extremely significantly lower Shannon–Wiener diversity index than PA-L and PA-H. The Simpson dominance index of PA-L and PA-H were significantly lower than SA-L and SA-H (*p* < 0.05), The Margalef richness index of PA-L and PA-H was significantly higher than ZL-L (*p* < 0.05) (Figure 2a). Except for the fact that the Margalef abundance index of PA-L and PA-H were significantly higher than those of SA-L and SA-H (*p* < 0.05) (Figure 2b), there was no significant difference in the species diversity between the various plots in the summer. The species diversity of the autumn plots was comparable to that of the spring: the Shannon–Wiener diversity index and Margalef abundance index of PA-L and PA-H were significantly higher than ZL-L (*p* < 0.05), and Pielou’s evenness index of PA-L and PA-H was extremely significantly higher than ZL-L (*p* < 0.01). ZL-L was significantly higher than PA-L and PA-H (*p* < 0.05) in terms of the Simpson dominance index (Figure 2c).

**Figure 3:**
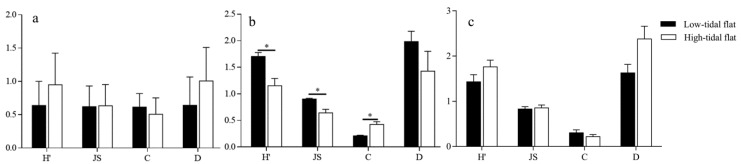
Comparison of the diversity indices of the different types of tidal flats in (**a**) spring, (**b**) summer, and (**c**) autumn. H’ = Shannon–Wiener index, JS = Pielou’s evenness index, C = Simpson dominance index, and D = Margalef abundance index. The values are the means ± SE. * indicates the level of significance. * = significant (*p* < 0.05).

The species diversity of the Collembola community in various tidal flat types and across several seasons was compared. The results show that, in contrast to the Simpson dominance index, the Shannon–Wiener diversity index and the Pielou’s evenness index of ZL-L, PA-L, and SA-L are both higher than PA-H and SA-H (*p* < 0.05) in spring (Figure 3a). In the summer and autumn, there is no significant difference between the various types of tidal flats, but from the perspective of value, the species of the high-tidal flat are more diverse (Figure 3).

**Figure 4:**
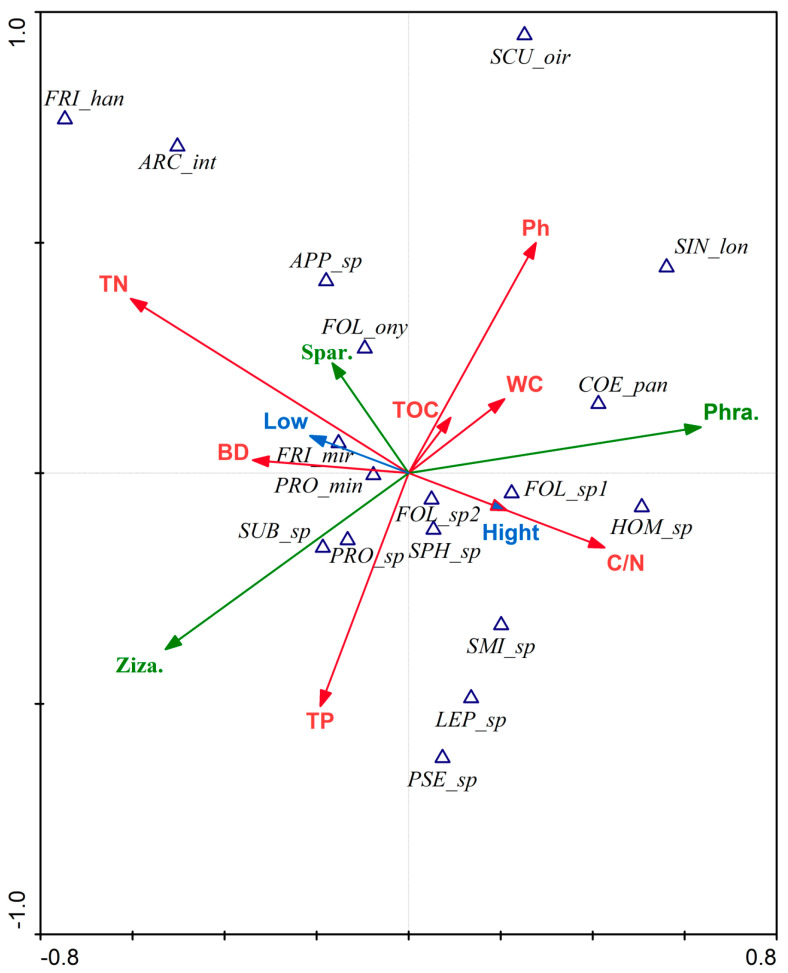
Parameter ordination of the environmental factor-species canonical correlation analysis. Triangle: species. Blue arrow: tidal flat, high tidal flat (High), and low tidal flat (Low). Green arrow: plants, *Zizania latifolia* (Ziza.), *Phragmites australis* (Phra.), and *Spartina alterniflora* (Spar.). Red arrow: physicochemical properties, water content (WC), bulk density (BD), power of hydrogen (Ph), total nitrogen (TN), total phosphorus (TP), total organic carbon (TOC), and carbon-nitrogen ratio (C/N).

Unconstrained ordination-DCA was performed on the data of the physicochemical properties of the soil environment, vegetation difference, tidal flat differences, and species composition. The highest value of the four axes was 3.086, between 3 and 4. CCA was used to investigate the correlation between environmental conditions and species. The variations explained by the two retrieved ranking axes were 61.9% and 73.4%, respectively. The results indicate that the bulk density positively correlates with *Proisotoma minuta,* the dominant species. *Proisotoma* sp. is positively correlated with the total P and *Zizania latifolia* and negatively correlated with the pH value. Among the common groups, *Subisoma* sp. is positively correlated with the total P and *Zizania latifolia,* and negatively correlated with the pH value. *Folsomides* sp. 1 is positively correlated with the high tidal flat and the C-N ratio, negatively correlated with the total N, and *Coecobrya pani* positively correlates with *Phragmites australis*. Among other species, *Homidia* sp.1 and *Folsomides* sp. 1 are positively correlated with the C-N ratio and negatively correlated with the total N (Figure 4).

### 3.4. Spatial and Temporal Distribution of the Functional Traits of Collembola

Four of the seven functional traits of Collembola that were examined are strongly related to the vertical distribution and seasonal changes.

#### 3.4.1. Body Length and Furcula Length

**Figure 5:**
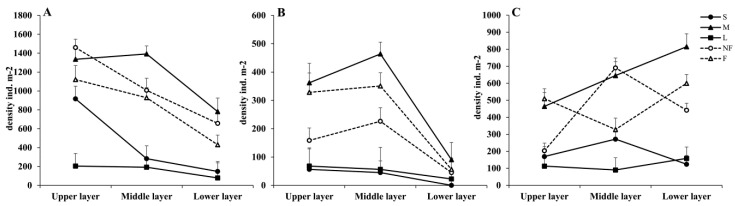
Vertical distribution of the body length and furcula length of the springtail in (**A**) spring, (**B**) summer, and (**C**) autumn. S = the small-sized Collembola, M = the medium-sized Collembola, L = the large-sized Collembola, NF = the Collembola with furcula < 0.1 mm, F = the Collembola with furcula > 0.1 mm. The values are the means ± SE.

The medium-sized Collembola (M) has the highest proportion in the spring and is mostly found in the upper and middle layers (*p* < 0.05). In contrast, the small-sized Collembola (S) is mostly found in the upper layer (*p* < 0.05), and the large-sized Collembola (L) has the lowest proportion and decreases as the soil depth increases (*p* < 0.05). Both Collembola with furcula <0.1 mm (NF) and Collembola with furcula >0.1 mm (F) are primarily found in the upper and mid-layers (*p* < 0.05), and their proportion decreases as soil depth increases (Figure 5A).

The proportion of medium-sized Collembola (M) increased in the summer and is still mostly distributed in the upper and middle layers than in the lower layer (*p* < 0.05). The proportion of Collembola with developed furcula (F) is relatively high and primarily distributed in the upper and middle layers (*p* < 0.05), similar to that of Collembola with undeveloped furcula (NF). The ratio of both groups is smaller in the upper layer than in the middle layer, which is an interesting finding (Figure 5B).

The distribution pattern in autumn is very different from spring and summer. Medium-sized Collembola continue to make up the largest proportion of body length, which rises gradually as the soil depth increases (*p* < 0.05). The second-largest proportion (middle layer > upper layer > lower layer) belongs to small-sized Collembola. Large-sized Collembola are distributed as follows: lower layer > upper layer > middle layer (*p* < 0.05). The distribution of the Collembola with undeveloped furcula (NF) is primarily in the middle layer (*p* < 0.05). In contrast, the distribution of the Collembola with developed furcula (F) is primarily in the upper and lower layers (Figure 5C).

#### 3.4.2. Sense-Related Organs: PAO and Antennae

**Figure 6:**
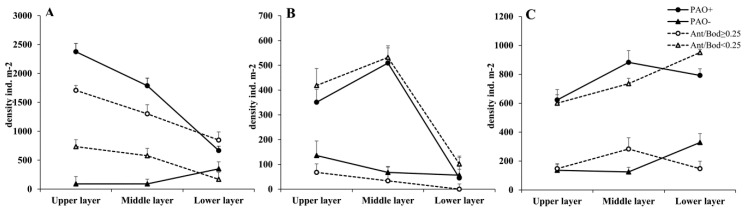
Vertical distribution of springtail’s PAO and antenna length/body length in (**A**) spring, (**B**) summer, and (**C**) autumn. PAO+ = Collembola with PAO, PAO−= Collembola without PAO, Ant/Bod ≥ 0.25 = Collembola with a long antenna (length ratio of antenna/body ≥ 0.25), Ant/Bod < 0.25 = Collembola with a short antenna (length ratio of antenna/body < 0.25). The values are the means ± SE.

In spring, there is more Collembola with PAO (PAO+) than without PAO (PAO−). The former group is mostly found in the upper and middle layers (*p* < 0.05), and as the soil depth increases, so does the proportion of this group. Collembola without PAO are primarily distributed in the lower layer (*p* < 0.05). When comparing Collembola, the proportion with a long antenna (Ant/Bod ≥ 0.25) is higher than the proportion with a short antenna (*p* < 0.05) (Ant/Bod < 0.25). Both groups follow the vertical distribution pattern: upper layer > middle layer > lower layer (*p* < 0.05) (Figure 6A).

The ratio of PAO+ is higher than PAO− in the summer. Similar to the springtime distribution pattern, PAO+ is primarily found in the upper and middle layers (*p* < 0.05). The distribution of Collembola with PAO is distinct from that in the spring because it is primarily found in the middle layer instead of the upper layer (*p* < 0.05). In contrast, Collembola without PAO are primarily found in the upper layer with a small proportion (*p* < 0.05). Additionally, Collembola without developed antennae make up a larger proportion and follow the same layer distribution as those with PAO: middle layer > upper layer > lower layer (*p* < 0.05). As the depth of the soil increases, a lower proportion of Collembola has developed antennae (Figure 6B).

The distribution law of sensory ability in autumn is significantly different from that in spring and summer. The distribution law of the PAO+ group is middle layer > lower layer > upper layer (*p* < 0.05). In contrast, the PAO− group is mostly distributed in the lower layer (*p* < 0.05), resulting in a larger proportion of Collembola with PAO than without PAO. As the soil depth increases, a greater percentage of Collembola have undeveloped antennae. The middle layer is where the majority of Collembola with PAO are found (Figure 6C).

### 3.5. Response Model of the Functional Traits of Collembola Diversity to the Environment

Unconstrained ordination-DCA was carried out on the data of the physicochemical properties of the soil environment, vegetation difference, and tidal flat differences to explore the response model of the functional traits of Collembola to the physicochemical properties of the soil environment in the sample plot. Four axes have a maximum value of 0.947, smaller than four. RDA was therefore chosen to investigate the relationship between environmental factors and functional qualities. The variations for environmental factors-functional qualities described by the two retrieved effective rankings axes are 34.9% and 50.9%, respectively.

**Figure 7:**
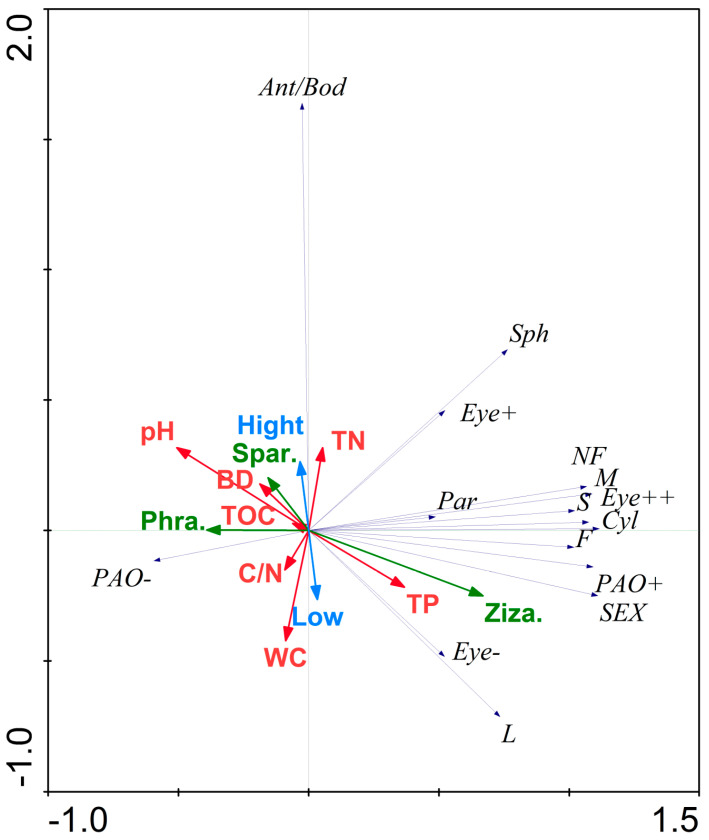
Parameter ordination of the environmental factor-species linear model-redundancy analysis. Blue arrow: tidal flat, high tidal flat (High), and low tidal flat (Low). Green arrow: plants, *Zizania latifolia* (Ziza.), *Phragmites australis* (Phra.), and *Spartina alterniflora* (Spar.). Red arrow: physicochemical properties, water content (WC), bulk density (BD), power of hydrogen (pH), total nitrogen (TN), total phosphorus (TP), total organic carbon (TOC), and carbon-nitrogen ratio (C/N). Thin Blue arrow: functional traits.

The findings demonstrate that the length ratio of antenna/body was positively correlated with the total N and high tidal flat and negatively correlated with the soil water content and low tidal flat; the characteristic of PAO− is positively correlated with phragmites and organic C; the characteristic of Eye− is positively correlated with the total P of large-sized Collembola (L) and negatively correlated with the soil bulk density and pH value; the spherical shape is negatively correlated with the C-N ratio of soil; parthenogenesis is positively correlated with *zizania* and negatively correlated with the pH value; NF, small-, and medium-sized Collembola (S, M), traits of Eye+, and cylindrical shape are positively correlated with phragmites and organic C (Figure 7).

## 4. Discussion

### 4.1. Analysis of the Physicochemical Properties of the Soil

The soil physicochemical properties in the environment show that the types of vegetation and the difference between high and low tidal flats impact those qualities. This is consistent with many studies that show that native species are more helpful in maintaining soil moisture than invasive species under similar vegetation height and density [39,45,46]. The soil water content of the sample plot, whose main vegetation is *Phragmites australis,* is significantly higher than that of *Spartina alterniflora* (*p* < 0.05). The pH level is higher at the high tidal flats. A study about the spatiotemporal characteristics of landscape change in coastal wetlands is comparable to this one [47]. Once the high tidal flats are submerged by seawater, the interval between inundation grows longer, and more inorganic salts precipitate, increasing the salinity of the high tidal flat and affecting the outcome of acidity and alkalinity.

The total N value of *Spartina alterniflora* plots is higher than that of *Phragmites australis* plots, and the organic C value of *Spartina alterniflora* plots is lower than that of *Phragmites australis* plots, regardless of the level of tidal flats. The invasion of *Spartina alterniflora* increased the proportion of nematodes, especially the proportion of bacteria-eating nematodes, and altered the quality of the litter, according to a study on soil nematodes in Jiuduansha wetland [46]. By accelerating the decomposition of litter, *Spartina alterniflora* accelerated the transfer of N from the litter to the environment, and finally increased the total N.

Studies show that *Spartina alterniflora* is more likely to use the C absorbed by the aboveground stems and leaves during the process of using and distributing organic C, accelerating plant growth and improving the efficiency of *Spartina alterniflora* in absorbing organic C from the environment [48,49]. The underground portion of *Spartina alterniflora* is more likely to use the C absorbed by *Spartina alterniflora* when it receives C from the atmosphere. This also contributes to the decreased organic C content in *Spartina alterniflora* plots by transferring more C to microorganisms with a higher turnover efficiency.

### 4.2. Response Model of Springtail Species Diversity to the Environment

Jiuduansha wetland is one of the tidal flat wetlands in the Yangtze River Delta with the least human disturbance. It has fewer Collembolan species than inland-wetland ecosystems with longer succession times [50,51]. It had only one type and level of vegetation at the time of the field investigation, which contributed to the instability of the ecosystem and relatively poor species diversity. This lack of tree layer and shrub layer vegetation also indicated that the succession level of the sample plot is lower than the forest ecosystem.

The two dominant species, *Proisotoma minuta* and *Proisotoma* sp. are among the five species found in each sample plot, demonstrating their high adaptability to the Jiuduansha environment. *Proisotoma minuta*, a springtail, can be found in various ecological environments [52]. However, a study on Collembola in wetlands reveals that the dominant species were *Oligaphorura ursi* and *Tullbergia* sp.1 [53], which were unrelated to the Isotomidae. It was hypothesized that the causes were the dimensional differences and the influence of fresh water and seawater, which differed from our investigation findings.

The richest Collembolan species are found in the *Phragmites australis* plots, while the *Zizania latifolia* plot has the fewest species. A previous study on coastal wetlands is comparable to our research [54]. In terms of how vegetation types affect species diversity, the Shannon–Wiener diversity index of *Phragmites australis* plots in spring is significantly higher than that of *Zizania latifolia* plots (*p* < 0.01), and significantly higher than that of *Spartina alterniflora* plots (*p* < 0.05).

There are no significant differences in species diversity between the tidal flats in the summer and the autumn tidal flat. However, there is a considerable difference between the species diversity in the spring low tide flat sample plot and the high tide beach sample plot. Although the high-tide flat sample plot is less affected by the tide, in spring, dominating the mass reproduction of dominant groups encroaches on the ecological niche of other species and occupies a dominant position in competition with other species. The low-tide beach sample plot had a larger species variety in spring than the high-tide flat sample plot due to this slight tidal disturbance. This is consistent with the opinion that an appropriate variation in environmental conditions is favorable to species variety [55].

*Proisotoma minuta* showed a positive correlation with the soil bulk density in the CCA between the physicochemical features of the soil environment and the variations in vegetation, tidal flats, and species [56]. *Proisotoma minuta* has a survival benefit in an environment of low soil porosity, as shown by the positive correlation between *Proisotoma* sp. and the soil total P. Although vegetation, tidal flat type, and the properties of the C-N ratio, total N, bulk density, and total P are the primary environmental factors affecting species distribution, the specific response model of the springtail community to the environment cannot be elucidated.

### 4.3. Response Model of the Functional Traits Diversity of Springtails to the Environment

The ability of springtails to move and disperse varies depending on the season and soil depth, according to research on how the diversity of their functional traits responds to temporal and spatial patterns. First, according to the body length results, most medium-sized springtails are found in the upper and middle layers consistent with the vertical distribution of species in the previous study [18]. However, in autumn, although the number of medium-sized springtails still accounts for the largest proportion, its proportion gradually increases with the deepening of the soil layer. A possible explanation is that the environment’s ground surface breaks down when temperature decreases, and underground species are less negatively affected by the low temperature [57].

The small and medium-sized springtails make up a relatively small proportion of the population and decrease with the depth of the soil layer, except for the upper-layer small springtails, which make up a larger proportion in spring. This is in line with the previous theory that spring is the season with warmer soil conditions, which can increase the growth rate and reproductive rate for many springtail species, and reproduction results in a large number of small springtails and more larvae occupying the upper soil [58].

Another functional trait related to the movement and spread of springtails is whether or not the furcula is developed. This vertical distribution feature changes significantly throughout the year. Springtime sees a higher percentage of springtails of NF than those with developed furcula. The opposite is true in the summer. One inference is that while there are many larvae in spring and furcula development is incomplete, a significant proportion of springtails have undeveloped furcula. Another inference is that open, unstable, and stressful situations call for higher motility [59]. Springtails with developed furcula have a higher proportion of them because they can move and spread and are more capable to survive [60]. Most springtails in the upper and lower layers in autumn have developed furcula, whereas the majority in the middle layer have not. Further research is required to reveal the cause of this outcome, which may be related to soil porosity.

Concerning the relationship between the sensory ability of the springtail and the season as well as the soil depth, most springtails possess the PAO, indicating that the PAO can help to improve the springtail’s sensory capacity [20]. It is also important to note that the soil substratum contains an increasing percentage of springtails without the PAO. This might be because the springtail in the soil substratum has fewer opportunities to move around and has no need to sense the stable environment of the deep soil [61]. In terms of the ratio of the body length of the antenna, the proportion of springtails with undeveloped antennae is higher in summer and autumn, whereas the proportion of springtails with developed antennae is the largest in the spring. The only logical explanation is that in spring, when other predatory arthropod juveniles are breeding, the springtail can lower the probability of being preyed upon because it has developed antennas that can help better avoid natural enemies or sense their approach [62].

According to the RDA results of the physicochemical characteristics of the soil environment and plant cover, along with the variations in tidal flat and functional traits, the length of the antenna/body is positively correlated with the soil total nitrogen and high tidal flats and is negatively associated with the soil water content and low tidal flats. In that situation, a plausible explanation is that there are more opportunities for the springtail to migrate on the surface of the high tidal flat when it is briefly and infrequently submerged in seawater. Then, in such a situation, its highly developed sensory organ is advantageous [63]. In addition, the larger porosity, rather than the soil with low bulk density, can allow the huge springtail to pass through, explaining the negative association between the antenna/body, PAO, and soil bulk density [20].

## 5. Conclusions

The invasive species *Spartina alterniflora* has a greater capacity to absorb soil organic C than the native species and has a higher conversion efficiency when accelerating the migration of N from litter into the soil. Since the total N is higher in the *Spartina alterniflora* plot than in the *Phragmites australis* plot, the soil organic C content in the *Spartina alterniflora* sample plot is lower than that in the *Phragmites australis* plot. Protecting native vegetation is essential. Dominant Collembolan species belong to the family Isotomidae. In the *Phragmites australis* plot, species diversity and number are higher. Functional traits, such as whether or not the furcula is developed, are related to movement and spreading ability. Seasonal variations in the vertical distribution, particularly in the summer, suggest that the developed furcula may also aid springtail survival. The results of the vertical distribution of the functional traits related to the springtail sense abilities support two hypotheses: first, the proportion of Collembola without PAO that choose the lower soil layer as their habitat is higher because the physicochemical environment of deep soil layer is more stable; second, during the season when the number of natural enemies in the soil increases, the springtails with well-developed furcula have a higher probability of avoiding the threat of natural enemies, in order to flourish. Some ecological issues, such as species invasion, can be effectively explained using the functional trait and environment analysis method.

## Figures and Tables

**Table 1:** Basic details of the different plots.

Number	Shoal	Plants	Tidal Flat	Coordinate	Code
1	Jiangyanansha	*Zizania latifolia*	Low tidal flat	31°13.7833′ N 121°54.2047′ E	ZL-L
2	Shangsha	*Phragmites australis*	Low tidal flat	31°13.7833′ N 121°54.2047′ E	PA-L
3	Shangsha	*Phragmites australis*	High tidal flat	31°13.7828′ N 121°54.2033′ E	PA-H
4	Zhongxiasha	*Spartina alterniflora*	Low tidal flat	31°13.7833′ N 121°54.2047′ E	SA-L
5	Zhongxiasha	*Spartina alterniflora*	High tidal flat	31°10.6453′ N 121°57.8279′ E	SA-H

**Table 2:** Functional traits of Collembola.

Traits	Functional Traits	Types of Data for RLQ and Models	Abbreviation
Habitus	Body shape	1: Cylindrical body	Cyl
		2: Spherical body	Sph
	Body length	1: <0.5 mm	S
		2: 0.5–1 mm	M
		3: >1 mm	L
Locomotory organs	Furcula length	1: >0.1 mm	F
		0: <0.1 mm	NF
Sensory or sense-related organs	Ocelli number	2: >5 1: 1–5	Eye++ Eye+
		0: Absent	Eye−
	PAO	1: Present	PAO+
		0: Absent	PAO−
	Antenna length	1: Long, length ratio of antenna/body ≥ 0.25	Ant/Bod ≥ 0.25
		2: Short, length ratio of antenna/body < 0.25	Ant/Bod < 0.25
Reproduction	Reproduction mode	1: Sexual reproduction	Sex
		2: Parthenogenesis	Par

**Table 3:** Physicochemical properties of the soil at different sites.

Code	Water Content (WC) (%)	Bulk Density (BD) (g/cm^3^)	pH	Total Nitrogen (TN) (g/kg)	Total Phosphorus (TP) (g/kg)	Total Organic Carbon (TOC) (g/kg)	Carbon-Nitrogen Ratio (C/N)
ZL-L	26.408 a (0.211)	1.017 ab (0.146)	7.94 b (0.968)	0.924 ab (0.028)	0.560 a (0.038)	12.150 a (0.911)	13.119 a (0.583)
PA-L	24.866 a (0.622)	0.974 b (0.237)	8.34 ab (0.977)	1.036 a (0.143)	0.523 ab (0.029)	15.431 a (2.706)	15.609 a (3.674)
PA-H	26.441 a (0.766)	1.083 a (0.226)	8.43 a (0.145)	0.816 ab (0.018)	0.521 ab (0.019)	26.168 a (14.487)	32.395 a (6.182)
SA-L	25.719 b (0.304)	1.098 a (0.409)	8.33 ab (0.110)	1.074 a (0.179)	0.494 ab (0.011)	13.593 a (0.907)	13.339 a (2.287)
SA-H	22.368 b (1.657)	1.050 ab (0.247)	8.61 a (0.393)	1.087 a (0.269)	0.483 b (0.013)	9.998 a (0.842)	10.592 a (2.812)

Each value (mean ± SE) represents the average of three replicates in different plots. Each value within parentheses represents the SE value of three replicates. Different lowercase letters within a column indicate a significant effect among the plots (*p* < 0.05).

**Table 4:** Species composition of Collembola in the Jiuduansha wetland.

Family	Taxon	Abbreviation	ZL-L		PA-L		PA-H		SA-L		SA-H		Total	
			Specimens	Percentage (%)	Specimens	Percentage (%)	Specimens	Percentage (%)	Specimens	Percentage (%)	Specimens	Percentage (%)	Specimens	Percentage (%)
Isotomidae	*Proisotoma minuta* (Tullberg, 1871)	PRO_min	255	56.67	14	23.73	31	34.07	32	44.44	90	50.28	422	49.59
	*Proisotoma* sp.	PRO_sp	141	31.33	5	8.47	16	17.58	9	12.50	41	22.91	212	24.91
	*Subisotoma* sp.	SUB_sp	43	9.56	6	10.17	5	5.49	3	4.17	8	4.47	65	7.64
	*Folsomides* sp. 1	FOL_sp1	2	0.44	11	18.64	6	6.59	12	16.67	19	10.61	50	5.88
	*Folsomides* sp. 2	FOL_sp2	0	0	0	0	2	2.20	0	0	3	1.68	5	0.59
	*Appendisotoma* sp.	APP_sp	0	0	1	1.69	1	1.10	2	2.78	3	1.68	7	0.82
	*Archisotoma interstitialis* Delamare-debouteville, 1953	ARC_int	1	0.22	0	0	0	0	2	2.78	1	0.56	4	0.47
	*Folsomina onychiurina* Denis, 1931	FOL_ony	1	0.22	1	1.69	0	0	0	0	0	0	2	0.24
	*Scutisotoma oirota* (Vilkamaa, 1988)	SCU_oir	0	0	1	1.69	0	0	0	0	0	0	1	0.12
Entomobryidae	*Coecobrya pani* Xu, Yu & Zhang, 2012	COE_pan	0	0	10	16.95	12	13.19	8	11.11	7	3.91	37	4.35
	*Sinella longiantenna* Zhang & Deharveng, 2011	SIN_lon	0	0	2	3.39	6	6.59	0	0	0	0	8	0.94
	*Homidia* sp.	HOM_sp	0	0	2	3.39	2	2.20	1	1.39	0	0	5	0.59
	*Lepidocyrtus* sp.	LEP_sp	0	0	0	0	1	1.10	1	1.39	0	0	2	0.24
	*Pseudosinella* sp.	PSE_sp	0	0	0	0	1	1.10	0	0	0	0	1	0.12
Neanuridae	*Friesea mirabilis* (Tullberg, 1871)	FRI_mir	0	0	2	3.39	2	2.20	2	2.78	0	0	6	0.71
	*Friesea handschini* Kseneman, 1938	FRI_han	0	0	0	0	0	0	0	0	1	0.56	1	0.12
Sminthurididae	*Sphaeridia* sp.	SPH_sp	7	1.56	4	6.78	6	6.59	0	0	4	2.23	21	2.47
	*Sminthurides* sp.	SMI_sp	0	0	0	0	0	0	0	0	2	1.12	2	0.24
		total	450		59		91		72		179		851	

## Data Availability

The datasets generated and analyzed in the present study may be available from the corresponding author upon reasonable request.

## Supplementary Materials

The following supporting information can be downloaded at: https://www.mdpi.com/article/10.3390/insects14020210/s1, Table S1: Collembola extraction data table; Table S2: ANOVA table of F- and *p*-values of linear mixed effects models on the effects of invasive plant species (plant; native plant species and invasive species) and tidal flats (tidal flat; low-tidal flats and high-tidal flats) on the soil properties.
