# Supplementary material for: Effects of Species Invasion and Inundation on the Collembola Community in Coastal Mudflat Wetland from the Perspective of Functional Traits
Source: Insects. 2023 Feb 19;14(2):210. doi: 10.3390/insects14020210 (PMC9963046; doi:10.3390/insects14020210)
Supplement: Supplementary file 1 [file insects-14-00210-s001.zip › Table S2.pdf]

Table S2. ANOVA table of F- and p-values of linear mixed effects models on the effects of invasive plant species (plant; native plant species and invasive species) and tidal flats (tidal flat; low-tidal flats and high-tidal flats) on the soil properties. Significant effects (P < 0.05) are given in bold.

	WC		BD		pH		TN		TP		TOC		C/N	
	F-value	p-value	F-value	p-value	F-value	p-value	F-value	p-value	F-value	p-value	F-value	p-value	F-value	p-value
Tidal flat	0.82	0.39	1.26	0.29	**5.74**	**0.04**	0.34	0.57	0.11	0.75	**6.76**	**0.03**	1.33	0.28
plant	1.81	0.22	0.56	0.48	1.09	0.33	0.76	0.41	2.96	0.12	0.16	0.70	0.36	0.56
Tidal flat ×plant	2.09	0.19	**6.35**	**0.04**	1.49	0.26	0.43	0.53	0.05	0.82	0.37	0.56	0.04	0.85
